# Targetoid-like necrotic plaque after low–molecular-weight heparin injection

**DOI:** 10.1016/j.jdcr.2026.06.031

**Published:** 2026-06-22

**Authors:** Federico Sandoval Morales, Anaïs Cauliez, François Aubin

**Affiliations:** a Dermatology and Allergy Department, University Hospital, Besançon, France; b Inserm Right 1098, Marie et Louis Pasteur University, Besançon, France

**Keywords:** enoxaparin, heparin-induced, heparin-induced thrombocytopenia, low–molecular-weight heparin, skin necrosis, targetoid plaque

## Case description

A 70-year-old woman receiving apixaban was hospitalized following a traumatic fall complicated by rib fractures and hemothorax. Oral anticoagulation was temporarily discontinued and replaced with subcutaneous enoxaparin.

Five days after the initiation of enoxaparin, she developed a painful lesion at an abdominal injection site. Physical examination revealed a sharply demarcated erythematous targetoid plaque characterized by a central violaceous area surrounded by an erythematous halo (Fig 1, *A*). The lesion progressed to cutaneous necrosis with eschar formation within 7 days (Fig 1, *B*). No additional skin lesions were observed.

**Fig 1:**
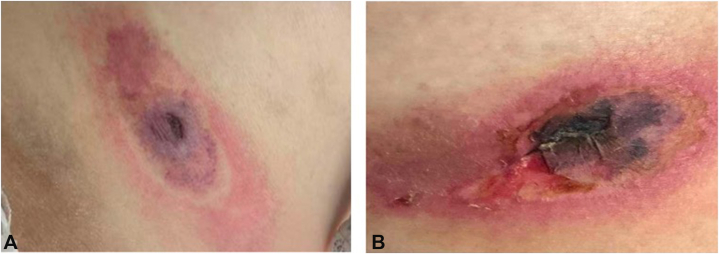
Clinical progression of enoxaparin-induced skin necrosis. **A,** Sharply demarcated targetoid erythematous plaque with a central violaceous ecchymotic area at the abdominal injection site. **B,** Progression to cutaneous necrosis with central eschar formation 7 days after lesion onset.

Laboratory testing showed a decrease in platelet count from 286 × 10^9^ g/L to 145 × 10^9^ g/L (49% decrease) by day 5. Anti–platelet factor 4/heparin antibodies were negative.

Enoxaparin was discontinued, and oral anticoagulation was resumed. The platelet count normalized 4 days after the discontinuation of enoxaparin. The lesion improved progressively with local wound care alone, without surgical intervention. The resulting ulcer healed progressively over several months with local wound care, leaving residual scarring.

**Question 1: What is the most likely diagnosis?**

**A.** Heparin-induced skin necrosis (non–heparin-induced thrombocytopenia [HIT] mediated)
**B.** Heparin-induced thrombocytopenia–associated necrosis
**C.** Nicolau syndrome (embolia cutis medicamentosa)
**D.** Calciphylaxis
**E.** Cutaneous small-vessel vasculitis

**Answer:**

**Correct answer:** **A.** Heparin-induced skin necrosis (non–HIT mediated). Heparin-induced skin necrosis is a rare complication associated with both unfractionated and low–molecular-weight heparins. It typically develops within 5 to 14 days after initiation and presents at injection sites as a painful, well-demarcated erythematous plaque with a targetoid appearance and central violaceous discoloration, progressing to necrosis and eschar formation.^1^^,^^2^

Two pathogenic mechanisms have been described. The immune-mediated form is associated with type II HIT, in which anti-PF4/heparin antibodies induce platelet activation, leading to microvascular thrombosis and tissue necrosis. In contrast, non–HIT-mediated forms are characterized by absent or mild thrombocytopenia and negative anti-PF4/heparin antibodies. Local vascular injury, microthrombosis, or direct cytotoxic effects at the injection site are thought to predominate in these cases.

In the present case, negative anti-PF4 antibodies, mild thrombocytopenia, and favorable evolution after discontinuation of enoxaparin, without clinical evidence of systemic thromboembolic complications, are most consistent with a non–HIT-mediated mechanism.

Diagnosis is primarily clinical, based on characteristic lesion morphology and temporal association with heparin exposure. Early recognition is essential, as prompt withdrawal of heparin typically prevents progression and systemic complications.

## Conflicts of interest

None disclosed.
